# Telmisartan cardioprotects from the ischaemic/hypoxic damage through a miR‐1‐dependent pathway

**DOI:** 10.1111/jcmm.14534

**Published:** 2019-08-01

**Authors:** Maria Consiglia Trotta, Bartolo Ferraro, Antonietta Messina, Iacopo Panarese, Eliana Gulotta, Giovanni Francesco Nicoletti, Michele D’Amico, Gorizio Pieretti

**Affiliations:** ^1^ Department of Experimental Medicine University of Campania ‘Luigi Vanvitelli’ Naples Italy; ^2^ Department of Mental and Physical Health and Preventive Medicine University of Campania ‘Luigi Vanvitelli’ Naples Italy; ^3^ Department of Surgical, Oncological and Stomatological Disciplines University of Palermo Palermo Italy; ^4^ Multidisciplinary Department of Surgical and Dental Specialties University of Campania ‘Luigi Vanvitelli’ Naples Italy

**Keywords:** Bcl‐2, connexin 43, hypoxic H9c2 cells, KCNQ1, miR‐1, myocardial ischaemia/reperfusion, telmisartan

## Abstract

The aim of this study was to investigate whether telmisartan protects the heart from the ischaemia/reperfusion damage through a local microRNA‐1 modulation. Studies on the myocardial ischaemia/reperfusion injury in vivo and on the cardiomyocyte hypoxia/reoxygenation damage in vitro were done. In vivo, male Sprague‐Dawley rats administered for 3 weeks with telmisartan 12 mg/kg/d by gastric gavage underwent ischaemia/reperfusion of the left descending coronary artery. In these rats, infarct size measurement, ELISA, immunohistochemistry (IHC) and reverse transcriptase real‐time polymerase chain reaction showed that expressions of connexin 43, potassium voltage‐gated channel subfamily Q member 1 and the protein Bcl‐2 were significantly increased by telmisartan in the reperfused myocardium, paralleled by microRNA‐1 down‐regulation. In vitro, the transfection of cardiomyocytes with microRNA‐1 reduced the expressions of connexin 43, potassium voltage‐gated channel subfamily Q member 1 and Bcl‐2 in the cells. Telmisartan (50 µmol/L) 60 minutes before hypoxia/reoxygenation, while not affecting the levels of miR‐1 in transfected cells in normoxic condition, almost abolished the increment of miR‐1 induced by the hypoxia/reoxygenation to transfected cells. All together, telmisartan cardioprotected against the myocardial damage through the microRNA‐1 modulation, and consequent modifications of its downstream target connexin 43, potassium voltage‐gated channel subfamily Q member 1 and Bcl‐2.

## 1 INTRODUCTION

Myocardial hypoxia or ischaemia‐associated hypoxia is a major determinant of complex pathological changes in the heart, since a constant supply of oxygen is indispensable for cardiac viability and function.^1^ The hypoxic state leads to a continuous cardiomyocyte structural and electrophysiological remodelling.^2^, ^3^ This is characterized by modifications of the ATP‐sensitive potassium (KATP) channels, important sensors for cardiac action potential repolarization^3^, ^4^, ^5^, ^6^ and modifications of connexin 43 (Cnx43), the main constitutional protein of the gap junctions into the ventricle.^7^, ^8^ These modifications are induced through changes in the local expression of microRNA‐1 (miR‐1)^9^, ^10^ which targets both Cnx43 and the potassium voltage‐gated channel subfamily Q member 1 (KCNQ1 or Kv7.1),^11^, ^12^, ^13^, ^14^ even if their combined role is not fully understood.

Telmisartan, an angiotensin II type 1 (AT1) receptor blocker and partial peroxisome proliferator‐activated receptor gamma (PPAR‐*γ*) agonist, has recently shown protective effects in the treatment of hypoxic/ischaemic cardiac damage^15^, ^16^, ^17^ related to increased levels of cardiac Cnx43 and reduced apoptosis.^18^, ^19^ However, it has not yet been studied whether the effects of telmisartan on Cnx43 have been exerted through modifications of the expression of the cardiac miR‐1. Therefore, the first objective of the present study was to study the effects of telmisartan treatment on the cardiac expression of miR‐1, Cnx43 and KCNQ1 in a rat model of myocardial ischaemia/reperfusion (I/R) injury. Then, to better define the molecular mechanisms determined by telmisartan, the cardiac expression of miR‐1, Cnx43 and KCNQ1 was monitored in hypoxic/reoxygenated embryonic rat ventricle H9c2 cells. This in vitro setting was further used to evaluate the putative effects of telmisartan on the transfected miR‐1 levels and thus on both Cnx43 and KCNQ1 expression. Furthermore, the anti‐apoptotic protein Bcl‐2, a further target of the silencing activity of the miR‐1, was monitored.^20^

## 2 MATERIALS AND METHODS

### 2.1 Animal treatment and surgical procedure

All the experimental procedures were approved by the Animal Ethics Committee of University of Campania ‘Luigi Vanvitelli’ of Naples (Protocol Number 2109, 27/7/12). Animal care was in compliance with Italian (DL 116/92) and European Commission (OJ of EC L358/1 18/12/86) guidelines on the use and protection of laboratory animals. All efforts were made to reduce the number of animals used and to minimize animal suffering. Male Sprague‐Dawley rats (220 ± 15 g, n = 40 total) were fed with tap water ad libitum and a standard chow diet for 3 weeks. The animals were randomly divided into four experimental groups (n = 10 per group): (a) untreated rats subjected to thoracotomy only and used as control (SHAM group); (b) untreated rats subjected to myocardial ischaemia/reperfusion (I/R) injury (I/R group); (c) rats administered for 3 weeks by gastric gavage with vehicle (1% methylcellulose, 4 mL/kg/d) and then subjected to I/R injury (I/R veh group); and (d) rats administered for 3 weeks by gastric gavage with telmisartan 12 mg/kg/d (Boehringer Ingelheim) and then subjected to I/reperfusion injury (I/R Tel group). According to previous evidence, this dose was effective in experimental myocardial ischaemia/reperfusion (I/R) injury.^15^, ^16^ At the end of the 3 weeks, rats were anaesthetized with intraperitoneal urethane (1.2 g/kg) and underwent thoracotomy or myocardial injury (I/R), as previously described with modifications.^15^ Briefly, in order to permit artificial ventilation when required, the rats were subjected to tracheotomy by using a polythene cannula. Then, the left thoracotomy was performed between the fourth and the fifth ribs. After the pericardium removal, the heart was exteriorized and then a fine silk ligature was placed around the left anterior descending coronary artery (LADCA), close to its origin. After 25 minutes of ischaemia, a 2‐hour reperfusion was performed. Rats were kept under artificial ventilation with room air at a rate of 56 strokes/min, a stroke volume of 1.0‐1.5 mL/100 g and a positive‐end expiratory pressure of 0.5‐1 cm H_2_O. Overall mortality was <5% throughout the entire study, in line with the usual procedures in our laboratory.^21^

### 2.2 Measurement of area at risk and infarct size

After the 2‐hour reperfusion period, the ratios between the weights of area at risk (AR) and left ventricle (LV) (AR/LV), infarct size (IS) and area at risk (IS/AR) and infarct size and left ventricle (IS/LV) were measured with Evans Blue dye as previously described.^15^ Selected experiments (n = 5 for each experimental group) were repeated monitoring AR without the staining procedures to measure IS. After the AR collection, half of each specimen was immediately frozen in liquid nitrogen for biochemical analyses and the other half was fixed by immersion in 10% buffered formalin and paraffin‐embedded for IHC.

### 2.3 Immunohistochemistry

A xylene substitute (HEMO‐De; Fisher Scientific) was used to remove paraffin from tissue sections and then rehydrated with ethanol gradient washes. A 3% hydrogen peroxide aqueous solution was used to sequentially quench the tissue sections, blocked for 1 hour at room temperature with phosphate‐buffered saline (PBS) 6% non‐fat dry milk (Bio‐Rad) and then incubated with specific antibodies anti‐Cnx43 (diluted 1:100; Santa Cruz; Cat. No. sc‐271837) and anti‐KCNQ1 (diluted 1:200; LifeSpan Biosciences; Cat. No. LS‐C405102), according to the recommended dilutions. After PBS washes, sections were incubated with secondary biotin‐conjugated goat anti‐rabbit IgG and avidin–biotin peroxidase complex (diluted 1:200; DBA; Cat. No. BA‐1000). An expert pathologist, blinded to the experimental protocol, analysed the specimens (intra‐observer variability 6%). The antigen expression was automatically calculated by using Image program LEICA IM500 and LEICA QWIN statistic program. Five distinct preparations for each group of animals were carried out, by analysing in each of them 20 microscope fields, for a total area of 2.4755 e^+004^ at 400× magnifications.

### 2.4 Hypoxic H9c2 cell culture

Embryonic rat ventricle H9c2 (2‐1) cardiomyocytes (Sigma; Cat. No. 88092904) were grown at 37°C under an atmosphere of 5% CO_2_ in 5.5 mmol/L glucose Dulbecco's modified Eagle's medium (DMEM; Aurogene; Cat. No. AU‐L0101‐500), supplemented with 10% heat‐inactivated foetal bovine serum (FBS) (Aurogene; Cat. No. AU‐S181H‐500), 5% l‐glutamine (Aurogene; Cat. No. AU‐X0550‐100) and 5% penicillin–streptomycin solution (Aurogene; Cat. No. AU‐L0022‐100).^14^

Hypoxic conditions were established according to a previous method by exposing H9c2 cells to cobalt chloride (CoCl_2_; Sigma; Cat. No. 15862) 400 µmol/L for 6 hours (H/R group) in DMEM 10%.^22^, ^23^, ^24^, ^25^ Hypoxia was confirmed by Western blot analysis of HIF‐1α protein expression. In order to investigate the effects of telmisartan treatment on hypoxic conditions alone, and not in combination with the serum and glucose deprivation (SGD), following the 6‐hour hypoxia the medium with CoCl_2_ was replaced by DMEM without CoCl_2_ for 2‐hour reoxygenation.^26^, ^27^ Sixty minutes before the hypoxia/reoxygenation,^23^ cells were exposed to a dose of telmisartan (50 µmol/L; H/R Tel group) already reported as effective on H9c2 cells, and to its vehicle dimethyl sulfoxide (DMSO 1% in cell medium; H/R DMSO group).^18^ At the end of the hypoxia/reoxygenation period, cell morphology was observed with optic microscopy (Leica DMi1, Germany). According to previous experience, cells were seeded at 5 × 10^3^ cells/cm^2^ in 96‐well plates for the viability assay; at 1 × 10^6^ cells/cm^2^ in 10 cm cell culture dishes for total RNA and protein detection; and at 1 × 10^4^ cells/cm^2^ in 24‐well plates for immunofluorescence assay.^28^ Three independent experiments were performed. In a single experiment, each treatment was repeated three times.

### 2.5 Cell viability assay

3‐(4,5‐dimethylthiazol‐2‐yl)‐2,5‐diphenyltetrazolium bromide (MTT) assay was used to measure cell viability.^14^ Briefly, the addition of MTT solution (1:10 in culture medium) to each well was followed by a 3‐hour incubation at 37°C. Once MTT solution was removed, each well was washed for 20 minutes at room temperature with isopropanol‐HCl 0.2 N. A 96‐well plate reader (iMark; Bio‐Rad Laboratories) was used to detect the optical density (OD) values at 570 nm.

### 2.6 miR‐1 mimic transfection

Normoxic H9c2 cells were transfected with miR‐1 mimic (mim1) 5 nmol/L (Qiagen; Cat. No. MSY0003125) (mim1 group) or negative control (mim1‐ group), using Lipofectamine 2000 reagent (Life Technologies; Cat. No. 11668‐027) according to the manufacturer's protocol. After 24 hours of transfection, miR‐1‐transfected cells were exposed to hypoxia/reoxygenation alone (H/R mim1 group) and to DMSO 1% or telmisartan 50 µmol/L before hypoxia reoxygenation (respectively, H/R mim1 DMSO and H/R mim1 Tel groups).

### 2.7 qRT‐PCR

miRNA isolation was performed with the miRNeasy Mini Kit (Qiagen; Cat. No. 217004), according to the manufacturer's protocol ‘Purification of Total RNA, including Small RNAs, from Animal Cells'. In order to monitor the miRNA recovery efficiency and to normalize miRNA expression in the real‐time PCR analysis, 5 μL of Syn‐cel‐miRNA‐39 miScript miRNA Mimic 5 nmol/L (Qiagen; Cat. No. MSY0000010) was spiked into each sample, before nucleic acid preparation. RNA concentration and quality were determined with NanoDrop 2000c Spectrophotometer (Thermo Fisher Scientific). Mature miRNAs were converted to cDNA with miScript II RT kit (Qiagen; Cat. No. 218161). The CFX96 Touch^™^ Real‐Time PCR Detection System (Bio‐Rad Laboratories) was used to monitor miR‐1 levels (MIMAT0000416). Each reaction was carried out with SYBR Green PCR Kit (Qiagen; Cat. No. 218073) and specific miScript Primer Assays for miR‐1 (Qiagen; Cat. No. MS00012943) and Syn‐cel‐miR‐39 (Qiagen; Cat. No. MS00019789). Δ*C*t value was as Ct_miR‐1_ − Ct_miR‐39_ in order to obtain miR‐1 levels as 2^−ΔCt^. Fold change was then obtained as 2^−ΔΔCt^ and calculated as 2^−ΔCt^ of the treatment group/2^−ΔCt^ of the control group. For fold change greater than 1, fold regulations were reported equal to the fold change values. For fold changes lower than 1, fold regulations were reported as the negative inverse of the fold change values.

### 2.8 Protein isolation and quantization

Heart tissues were homogenized in RIPA lysis buffer (Sigma; Cat. No. R0278) including a protease inhibitor cocktail (Roche; Cat. No. 11873580001). H9c2 cells were washed with cold PBS (Aurogene; AU‐L0615), scraped in 150 µL of cold RIPA lysis buffer (Sigma; Cat. No. R0278) including a protease inhibitor cocktail (Roche; Cat. No. 1187358000) and centrifuged at 13 800 g for 10 minutes at 4°C, to purify the protein supernatants from nucleic acids.^14^ Total protein concentration was determined following the Bio‐Rad protein assay protocol (Bio‐Rad Laboratories; Cat. No. 500‐0006) and used for Western blotting and ELISAs.

### 2.9 Western blotting assay

Thirty microgram of protein sample was separated on a 12% separation gel, electrotransferred onto a polyvinylidenedifluoride (PVDF) membrane (Merck Millipore; Cat. No. IPFL10100) and then blocked for 1 hour at room temperature with 5% non‐fat dry milk (Euroclone; Cat. No. EMR180500). The following specific primary antibodies were used for the overnight incubation of the blots using the recommended dilutions: anti‐HIF‐1α (diluted 1:200; Santa Cruz; Cat. No. sc‐8711), anti‐Cnx43 (diluted 1:500; Santa Cruz; Cat. No. sc‐271837), anti‐KCNQ1 (diluted 1:200; LifeSpan Biosciences; Cat. No. LS‐C405102) and anti‐actin (diluted 1:500; Santa Cruz; Cat. No.sc‐8432). Blots were then incubated for 1 hour at room temperature with horseradish peroxidase‐conjugated secondary anti‐rabbit (diluted 1:2000; Santa Cruz; Cat. No. sc‐2004), anti‐goat (diluted 1:2000; Santa Cruz; Cat. No. sc‐2020) or antimouse (diluted 1:2000; Santa Cruz; Cat. No. sc‐2005) antibodies. The signal was detected using the VisionWorks^™^ LS Analysis Software (Analytik Jena AG) and then expressed as densitometric units (DU).

### 2.10 ELISAs

Troponin I as marker of myocardial infarction and Bcl‐2 protein levels were quantified by using the Rat Cardiac Troponin I (cTnI) ELISA Kit (MyBioSource; Cat. No. MBS727624) and the Rat B‐cell CLL/lymphoma 2, Bcl‐2 ELISA Kit (MyBioSource; Cat. No. MBS704498), according to the manufacturer's protocols.

### 2.11 Immunofluorescence

H9c2 cells were fixed with 4% paraformaldehyde and washed with PBS (Aurogene; Cat. No. AU‐L0615). In order to inhibit non‐specific antibody binding, cells were incubated for 30 minutes in blocking solution (1% BSA in PBS). Cells were then incubated overnight at 4°C with the following primary antibodies, diluted in PBS blocking buffer: anti‐Cnx43 (diluted 1:100; Santa Cruz; Cat. No. sc‐271837) and anti‐KCNQ1 (diluted 1:200; Bioss Inc; Cat. No. bs‐6760R). Specific antigens were located using a fluorescein isothiocyanate (FITC)‐conjugated anti‐rabbit (diluted 1:1000; Immunoreagents; Cat. No. GTXRB‐003‐D488N). After the counterstaining H9c2 cells with pentahydrate bisbenzimide (Hoechst 33258; Sigma; Cat. No. 23491‐45‐4) and mounting with 90% glycerol in PBS, immunofluorescence images were obtained using a fluorescence microscope (Leica) and a fluorescence confocal microscope (LSM 710 Zeiss). Leica FW4000 (Leica) and Zen Zeiss (Zeiss) softwares were used to analyse the images. An observer blinded to the treatment performed the labelling quantization, by calculating the percentage of positive cells/total cells counted. This was calculated in each microscope field as the mean of labelled positive cells/400 cells counted. Four different fields were analysed for each treatment. Only bisbenzimide‐counterstained cells were considered as positive profiles in order to avoid overcounting cells.

### 2.12 Statistical analysis

The results are presented as mean ± standard error of the mean (SEM) of five observations per group in vivo, and as mean ± SEM of nine observations in vitro. Statistical significance was determined using ANOVA followed by Bonferroni's test. A *P*‐value <.05 was considered significant to reject the null hypothesis.

## 3 RESULTS

### 3.1 Myocardial tissue damage after telmisartan administration

**Figure 1:**
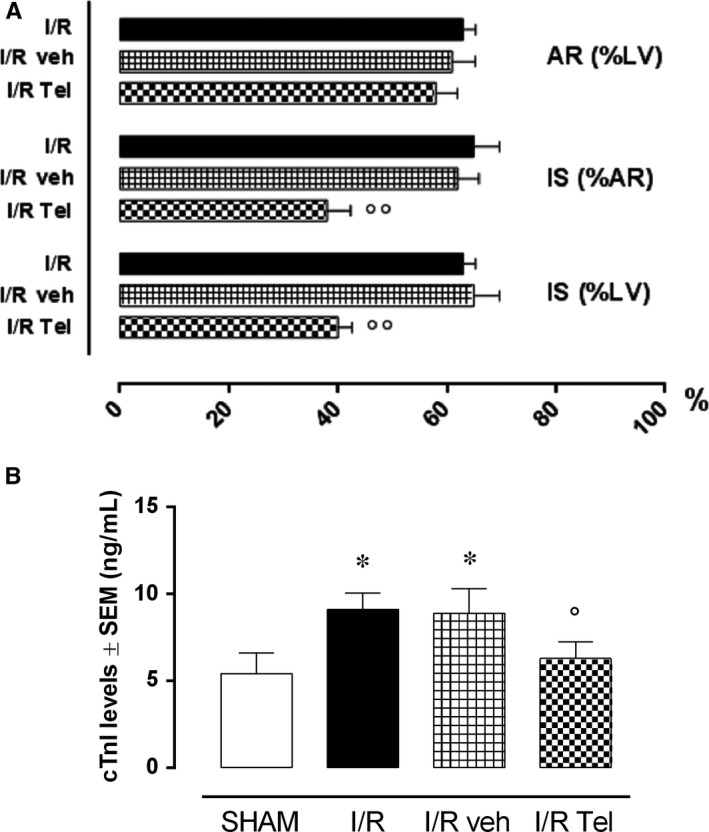
Effects of telmisartan on myocardial damage after I/R injury. A, AR/LV, IS/AR and IS/LV ratios and (B) cTnI levels in the different experimental groups. SHAM, thoracotomy only; I/R, ischaemia/reperfusion; veh, 1% methylcellulose 4 mL/kg/d; Tel, telmisartan 12 mg/kg; AR, area at risk; LV, left ventricle; IS, infarct size. Results are expressed as mean ± SEM of five observations. *P < .05 vs SHAM; °*P* < .05 and °°*P* < .01 vs I/R veh

Untreated Sprague‐Dawley rats subjected to I/R procedure (I/R group) exhibited an AR equal to the 63% of the LV. The IS was 65% of the AR and 40% of the LV (Figure 1A). The IS/AR values were not affected by the administration of vehicle (I/R veh) but were significantly reduced following treatment with telmisartan 12 mg/kg (I/R Tel; −38%, *P* < .01 vs I/R veh), as well as IS/LV ratios (−25%, *P* < .01 vs I/R veh; Figure 1A). Cardiac troponin I (cTnI) levels were significantly increased by I/R procedure (+67.9%, *P* < .05 vs SHAM) and significantly reduced in rats receiving telmisartan 12 mg/kg only (−31%, *P* < .05 vs I/R veh; Figure 1B).

### 3.2 Effects of telmisartan on Cnx 43, KCNQ1 and Bcl‐2 expression in I/R hearts

**Figure 2:**
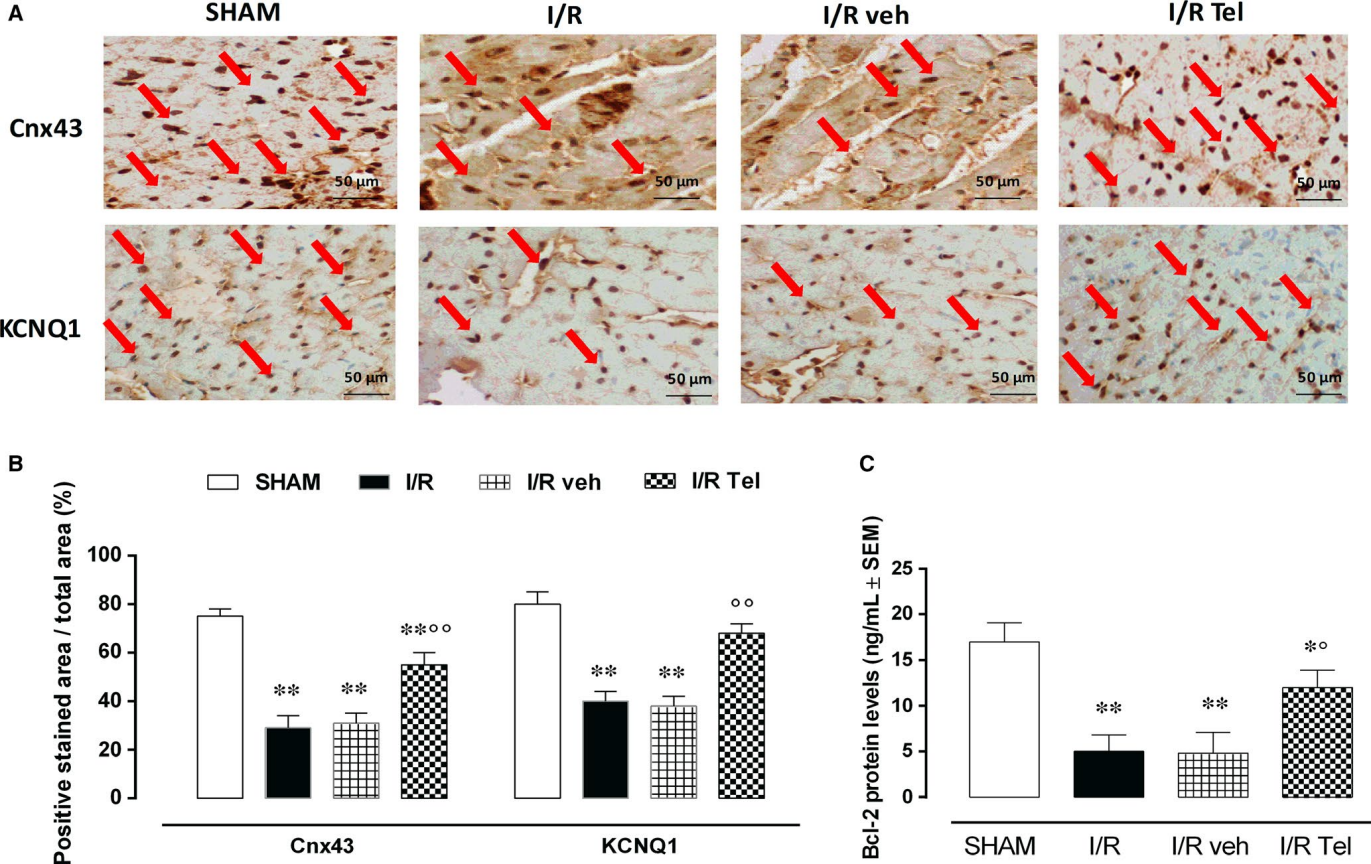
Effects of telmisartan on Cnx 43, KCNQ1 and Bcl‐2 expression in acute myocardial I/R injury. A, Representative images for immunochemical detection of Cnx43 and KCNQ1 levels. B, Bar graph showing Cnx43 and KCNQ1 expressed as percentage of positive stained area/total area. C, Bcl‐2 protein levels detected by ELISA. SHAM, thoracotomy only; I/R, ischaemia/reperfusion; veh, 1% methylcellulose 4 mL/kg/d; Tel, telmisartan 12 mg/kg. Results are expressed as mean ± SEM of five observations. **P < .01 vs SHAM; °*P* < .05 and °°*P* < .01 vs I/R veh. Scale bar = 50 µmol/L; 400× magnification

Following the I/R procedure, the IHC showed a significant reduction in Cnx43 and KCNQ1 expressions in I/R rats compared with SHAM group (respectively, −61.3% and −50%, *P* < .01 vs SHAM; Figure 2A,B). These were not significantly modified in I/R veh rats compared with I/R group (respectively, +6.9% and −5%), whereas I/R Tel group exhibited a consistent increase in both Cnx 43 and KCNQ1 levels compared with I/R group (respectively, +89.7% and +70%, *P* < .01 vs I/R veh; Figure 2A,B). The same trend was shown by Bcl‐2 levels, significantly decreased in I/R group (−70.6%, *P* < .01 vs SHAM; Figure 2C). These were markedly increased by telmisartan treatment +140% *P* < .05 vs I/R veh) and not by its vehicle (−4% vs I/R veh; Figure 2C).

### 3.3 miR‐1 modulation by telmisartan in I/R hearts

**Figure 3:**
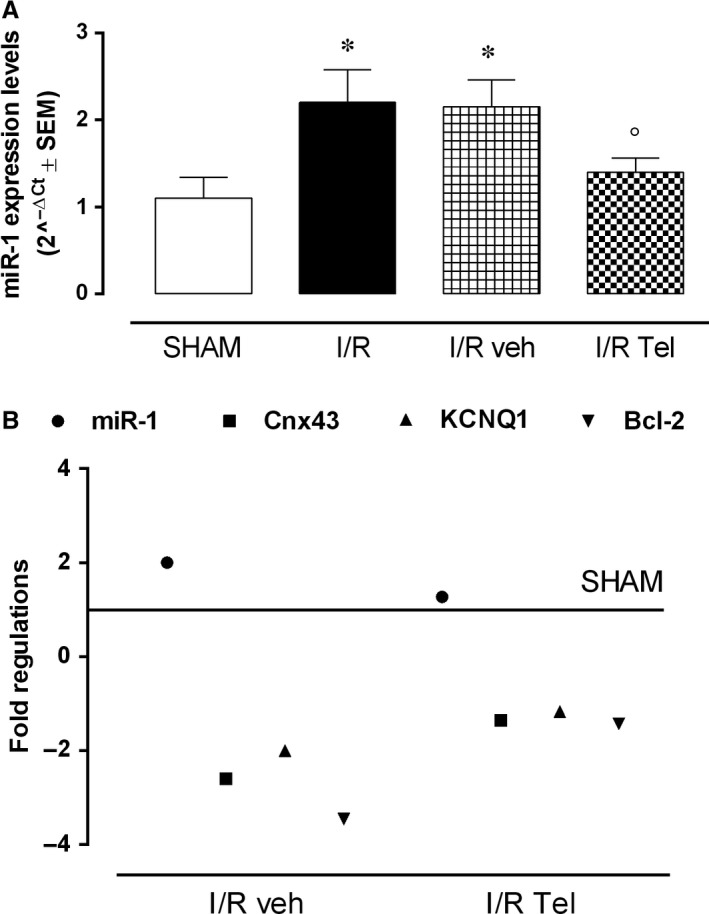
Effects of telmisartan on miR‐1 expression pattern in acute myocardial I/R injury. A, Bar graph showing qRT‐PCR miR‐1 levels expressed as 2^−ΔCt^ ± SEM (B) Fold regulation of miR‐1, Cnx43, KCNQ1 and Bcl‐2 in the different experimental groups. SHAM, thoracotomy only; I/R, ischaemia/reperfusion; veh, 1% methylcellulose 4 mL/kg/d; Tel, telmisartan 12 mg/kg. Results are expressed as mean ± SEM of five observations. *P < .05 vs Sham; °*P* < .05 vs I/R veh

qRT‐PCR analysis showed a significant increase in miR‐1 levels in rat hearts subjected to I/R procedure compared with SHAM rats (+100%, *P* < .05 vs SHAM; Figure 3A). Infarcted hearts exhibited an evident reduction in miR‐1 levels when administered with telmisartan 12 mg/kg (−36.4%, *P* < .05 vs I/R) and not with 1% methylcellulose vehicle (−2.3% vs I/R; Figure 3A). This miR‐1 down‐regulation was paralleled by an increased expression of Cnx43, KCNQ1 and Bcl‐2 in I/R Tel group (Figure 3B).

### 3.4 Cell morphology and viability in hypoxic/reoxygenated H9c2 cells exposed to telmisartan

**Figure 4:**
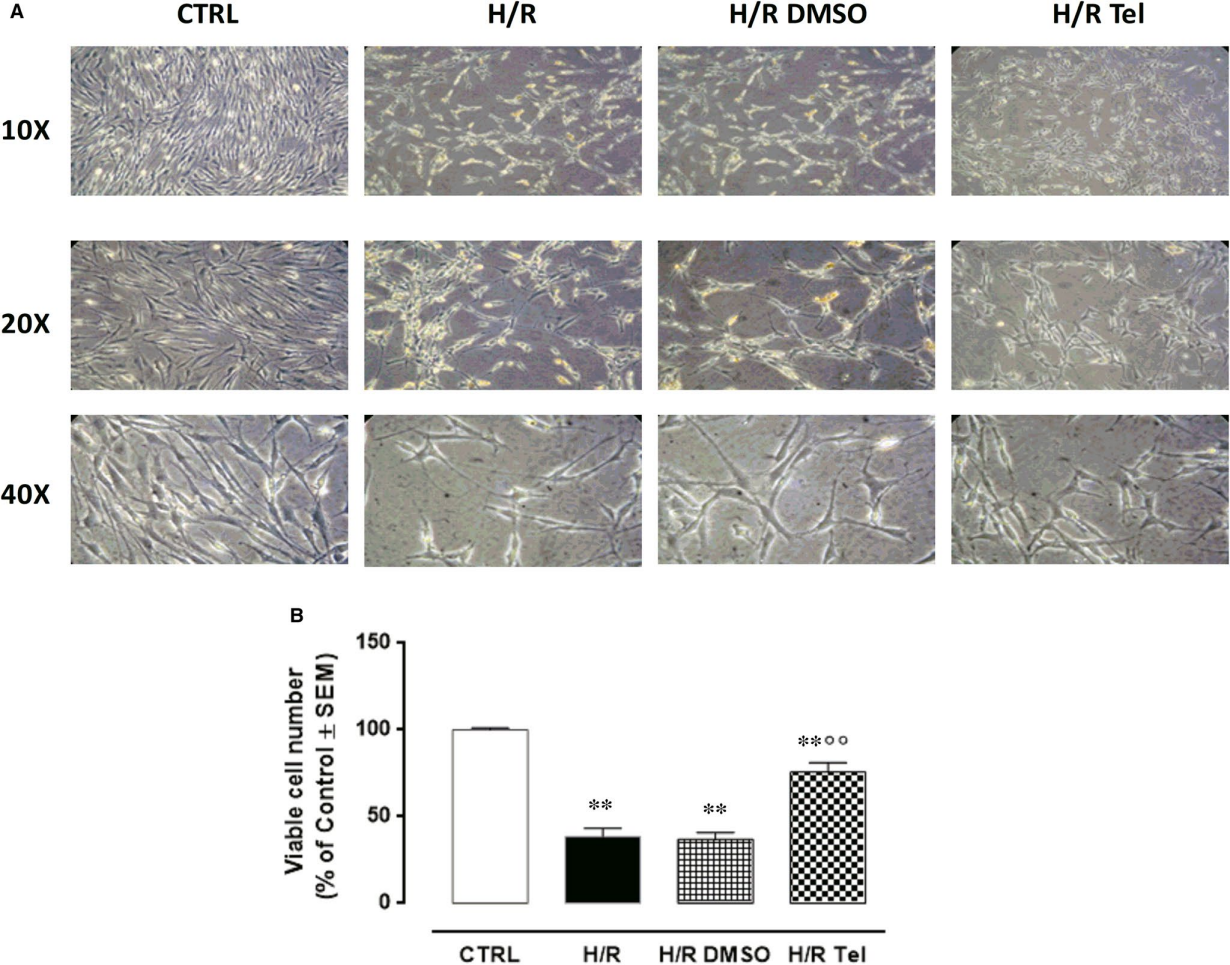
Effects of telmisartan on H/R H9c2 cell morphology and survival. A, Representative optical microscopy images at 10×, 20× and 40× magnifications. B, MTT assay for the determination of viable cell number. CTRL, normoxic cells; H/R, cells exposed to hypoxia/reoxygenation; H/R DMSO, H/R + DMSO 1%; H/R Tel, HR + telmisartan 50 µmol/L. Results are expressed as mean ± SEM of nine observations. **P < .01 vs CTRL; °°*P* < .01 vs H/R DMSO

H9c2 cells exposed to 400 µmol/L CoCl_2_ for 6 hours and to 2‐hour reoxygenation (H/R group) were severely damaged compared with normoxic cells (CTRL group), by exhibiting edge blurring, shedding, shrunken and floating in the cell culture medium (Figure 4A). They also had reduced cell viability (−61%, *P* < .01 vs CTRL) following the hypoxia/reoxygenation procedure (Figure 4B). Unmodified by DMSO exposure, the percentage of cell viability was significantly increased in H/R H9c2 cells pretreated with telmisartan 50 µmol/L (+96,4%, *P* < .01 vs H/R DMSO), although still reduced compared with CTRL (−24.4%, *P* < .01 vs CTRL) (Figure 4B). This was accompanied by a reduction in the damaged cell morphology (Figure 4A). Telmisartan and DMSO at the doses used did not modify the cell morphology and viability of normoxic H9c2 cells (data not shown).

### 3.5 Cnx 43, KCNQ1 and Bcl‐2 expression in H/R H9c2 cardiomyocytes

**Figure 5:**
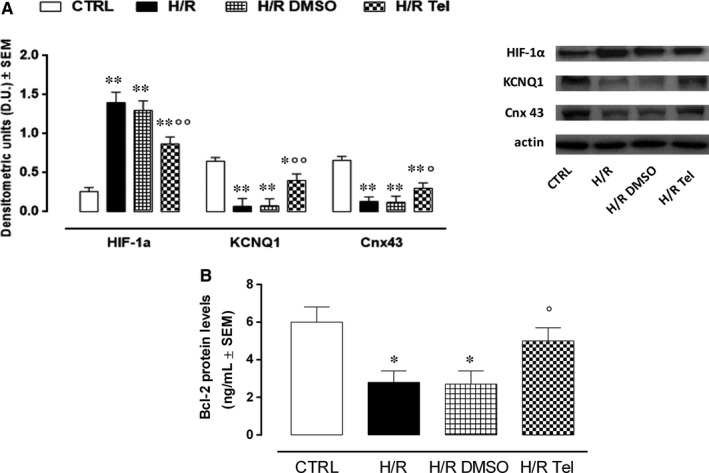
Modulation of Cnx43, KCNQ1 and Bcl‐2 by telmisartan in H7R H9c2 cells. A, Bar graph and representative Western blotting images, showing cell HIF‐1α, Cnx43 and KCNQ1 levels calculated as protein target/actin ratio. B, Bcl‐2 protein levels detected by ELISA. CTRL, normoxic cells; H/R, cells exposed to hypoxia/reoxygenation; H/R DMSO, H/R + DMSO 1%; H/R Tel, HR + telmisartan 50 µmol/L. Results are expressed as mean ± SEM of nine observations. *P < .05 and **P < .01 vs CTRL; °*P* < .05 and °°*P* < .01 vs H/R DMSO

Hypoxic conditions because of CoCl_2_ exposure were confirmed in H9c2 cells by detecting HIF‐1α cell protein levels through Western blotting analysis: This was significantly increased in all the experimental conditions compared with normoxic H9c2 cells (H/R, +438.5%; HR DMSO, +400.0%; H/R Tel, +234.6%, *P* < .01 vs CTRL; Figure 5A). Interestingly, telmisartan treatment (50 µmol/L) 60 minutes before hypoxia/reoxygenation model significantly reduced HIF‐1α levels in untreated hypoxic group (−37.9%, *P* < .01 vs H/R DMSO; Figure 5A). In addition to this, cells KCNQ1 and Cnx43 were found to be significantly reduced in H/R group (KCNQ1, −89.2%; Cnx43, −71.7%, *P* < .01 vs CTRL) compared with normoxic cells (Figure 5A). Also, Bcl‐2 protein levels showed this trend (−53.3%, *P* < .05 vs CTRL; Figure 5B). All these factors were not affected by DMSO pretreatment (respectively, +7.1%; −7.7% and −3.6% vs H/R DMSO), while markedly increased by telmisartan exposure (KCNQ1, +471.4%, *P* < .01 vs H/R DMSO; Cnx43, +130.8% and Bcl‐2, +325%, *P* < .05 vs H/R DMSO; Figure 5A,B). In contrast, both the vehicle DMSO and telmisartan at the doses used did not affect the factors monitored in normoxic H9c2 cells (data not shown).

### 3.6 miR‐1 modulation by telmisartan in hypoxic/reoxygenated H9c2

**Figure 6:**
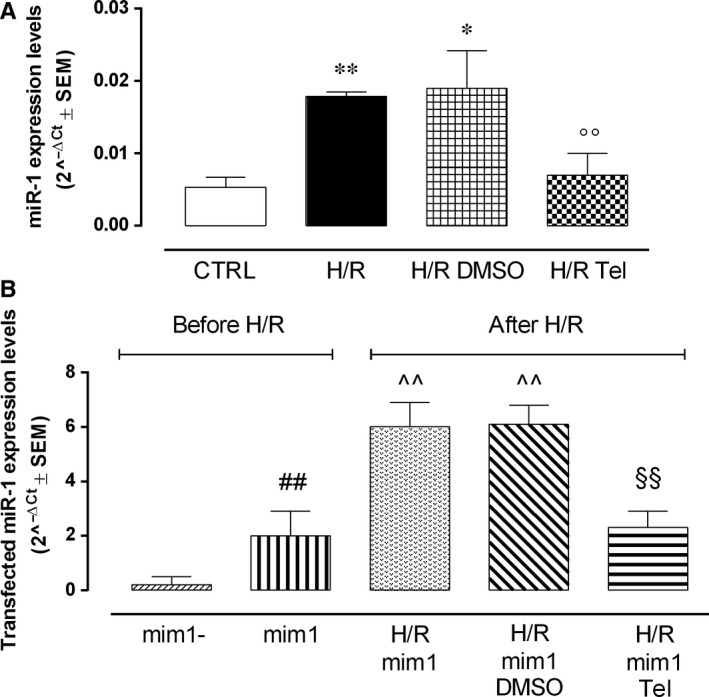
miR‐1 down‐regulation by telmisartan in myocardial hypoxia/reoxygenation in H9c2 cells. A, Bar graph showing qRT‐PCR miR‐1 levels expressed as 2^−ΔCt^ ± SEM in non‐transfected H9c2 cells. CTRL, normoxic cells; H/R, cells exposed to hypoxia/reoxygenation; H/R DMSO, H/R + DMSO 1%; H/R Tel, HR + telmisartan 50 µmol/L; B, transfected miR‐1 levels before and after hypoxia/reoxygenation, expressed as 2^−ΔCt^ ± SEM mim1‐, normoxic cells transfected with negative control mimic; mim1, normoxic cells transfected with miR‐1 mimic 5 nmol/L; H/R mim1, cells transfected with miR‐1 mimic 5 nmol/L and exposed to hypoxia/reoxygenation; H/R mim1 DMSO, H/R mim1 cells exposed to DMSO 1%; H/R mim1 Tel, H/R mim1 cells exposed to telmisartan 50 µmol/L. Results are expressed as mean ± SEM of nine observations. *P < .05 and **P < .01 vs CTRL; °°*P* < .01 vs H/R DMSO; ^##^ *P* < .01 vs mim1‐; ^^*P* < .01 vs mim1; ^§§^ *P* < .01 vs H/R mim1 DMSO

miR‐1 levels were strongly induced by hypoxia/reoxygenation in H9c2 cells (+237.7%, *P* < .01 vs CTRL), unmodified by DMSO (+6,1% vs H/R) and markedly reduced by telmisartan exposure (−63.2 *P* < .01 vs H/R DMSO) (Figure 6A).This drug did not produce any effect on miR‐1 in normoxic cells (data not shown).

### 3.7 miR‐1 modulation in miR‐1‐transfected hypoxic/reoxygenated H9c2

miR‐1 levels were quantified in miR‐1‐transfected cardiomyocytes. As expected, normoxic H9c2 cells transfected with miR‐1 mimic (mim+) exhibited significantly higher miR‐1 levels compared with cells transfected with the negative control (+900%, *P* < .05 vs mim1‐; Figure 6B). Hypoxia/reoxygenation further increased miR‐1 levels (+200%, *P* < .01 vs H/R mim1), as well as DMSO exposure before hypoxia/reoxygenation (205%, *P* < .01 vs H/R mim1). Interestingly, telmisartan while not affecting the levels of miR‐1 in transfected cells in normoxic condition, it almost abolished the increment of miR‐1 induced by the H/R procedure to these cells (−62.3%, *P* < .01 vs H/R mim1 DMSO; Figure 6B).

### 3.8 Cnx43, KCNQ1 and Bcl‐2 modulation by telmisartan in miR‐1‐transfected cardiomyocytes exposed to hypoxia/reoxygenation

**Figure 7:**
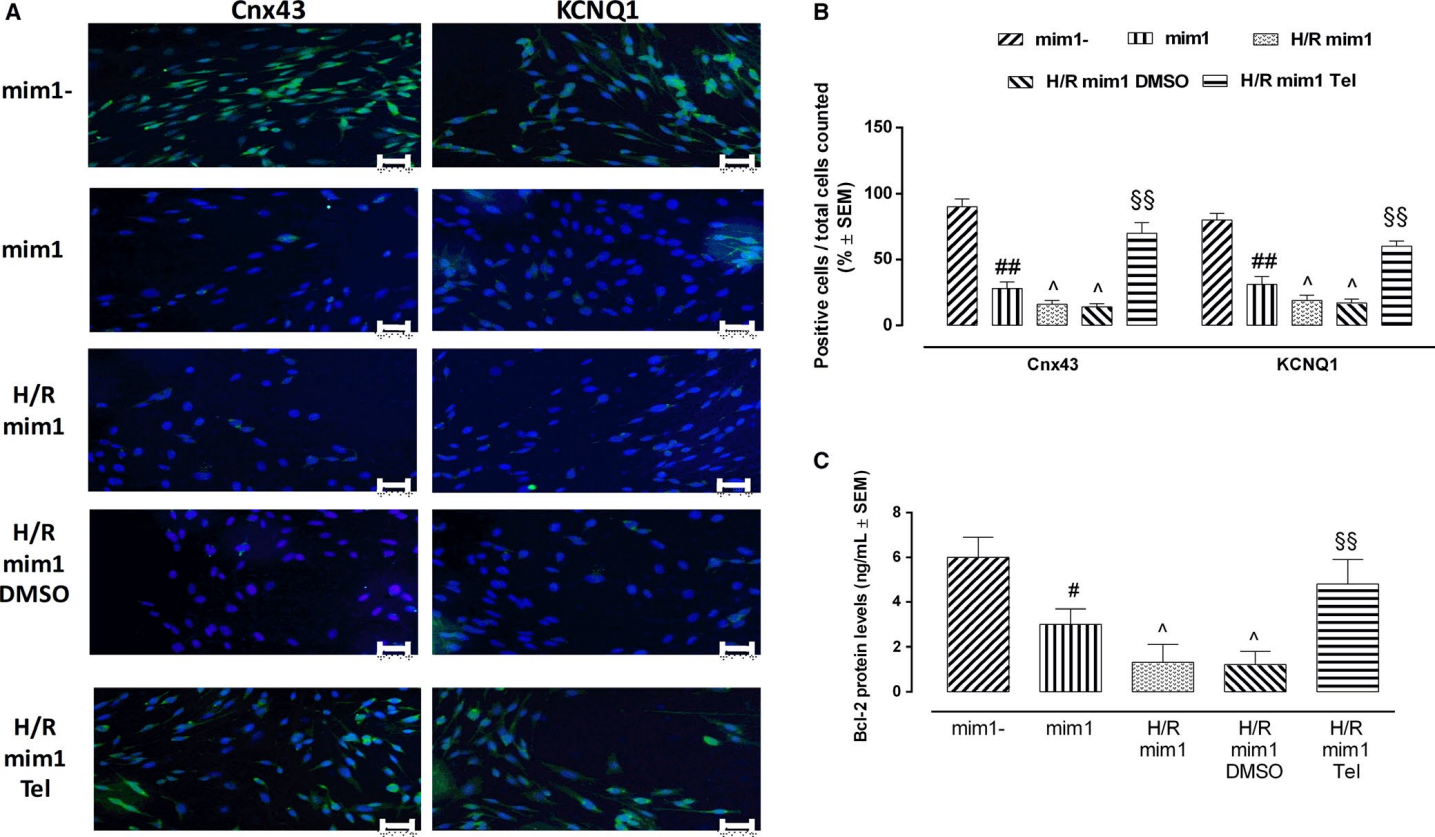
Telmisartan effects on hypoxic H9c2 cardiomyocytes transfected with miR‐1 mimic. A, Representative immunofluorescence images, showing Cnx3 and KCNQ1 levels in miR‐1‐transfected cells exposed to hypoxia/reperfusion. Cell nuclei are labelled in blue with Hoechst, whereas cells positive to Cnx43 or KCNQ1 antibodies are labelled in green. B, Bar graph showing the percentage of Cnx or KCNQ1‐positive cells/total counted cells. C, Bcl‐2 protein levels in miR‐1‐transfected H/R cardiomyocytes, detected by ELISA. mim1‐, normoxic cells transfected with negative control mimic; mim1, normoxic cells transfected with miR‐1 mimic 5 nmol/L; H/R mim1, cells transfected with miR‐1 mimic 5 nmol/L and exposed to hypoxia/reoxygenation; H/R mim1 DMSO, H/R mim1 cells exposed to DMSO 1%; H/R mim1 Tel, H/R mim1 cells exposed to telmisartan 50 µmol/L. Results are expressed as mean ± SEM of nine observations. ^#^ *P* < .05 vs mim1‐; ^##^ *P* < 0.01 vs mim1‐;^*P* < .05 vs mim1; ^§§^ *P* < .01 vs H/R mim1 DMSO. Scale bar = 10 µmol/L; 20× magnification

After hypoxia/reoxygenation, immunofluorescence analysis of miR‐1‐transfected H9c2 cardiomyocytes showed that telmisartan treatment caused a marked increase in Cnx43 (+400%, *P* < .01 vs H/R mim1 DMSO) and KCNQ1 compared with transfected cells unexposed to telmisartan (+253%, *P* < .01 vs H/R mim1 DMSO) (Figure 7A,B). In line with these, Bcl‐2 protein levels were significantly increased by telmisartan (+300%, *P* < .01 vs H/R mim1 DMSO) (Figure 7C).

## 4 DISCUSSION

Here, we report that telmisartan protected the infarcted rat heart by locally increasing two important players in hypoxia‐induced cell survival,^29^ Cnx43 and KCNQ1 potassium channel. In contrast, telmisartan reduced miR‐1 expression within the infarcted heart along with increased expression of the known apoptotic marker^20^, ^30^ B‐cell lymphoma 2 (Bcl‐2). This action resulted in a lower degree of tissue damage. Telmisartan also copied this effect on embryonic rat ventricle H9c2 cells exposed to a hypoxia/reoxygenation procedure.

The cardioprotection afforded by telmisartan has been widely reported in myocardial I/R injury^15^, ^16^, ^17^, ^18^, ^19^, ^31^; however, the present study for the first time sheds light on the involvement of the potassium channel KCNQ1 and Cnx43 with respect to telmisartan action. As it modulates cardiac cell susceptibility to hypoxia^32^, ^33^ and regulates the impulse propagation and electrical synchronization between cardiomyocytes,^34^, ^35^ Cnx43 seems to have a key role in the setting of functional I/R damage. Similarly, recent evidence reported that Cnx43 together with KCNQ1 is part of the same signalling pathway that provides cell protection after myocardial infarction by acting in a functionally dependent manner.^3^, ^29^, ^36^ Indeed, mutations in both genes encoding for KCNQ1 and Cnx43 have been associated with clinical sudden infant death syndrome (SIDS), for which hypoxia is a major risk factor.^37^, ^38^ The data presented, therefore, identified telmisartan as a regulatory candidate of these two important factors in the infarcted myocardium, Cnx43 and KCNQ1. Moreover, they highlighted that telmisartan cardioprotection is exerted by reducing the apoptosis within the myocardium relating to tissue protection. Indeed, apoptosis of cardiomyocytes, associated with quantitative disorders of proteins, aggravates myocardial I/R injury.^39^ It should also be noted that telmisartan decreased the resident miR‐1, which controls both the cardiac structure and the functionality. miR‐1 predominantly regulates the electrical and contractile activity of the heart, by modulating atrioventricular and ventricular conduction at multiple levels.^40^, ^41^, ^42^ Alterations of its expression levels result in atrioventricular block, impaired contractile function and increase in ROS levels following cardiac I/R injury.^9^, ^43^, ^44^, ^45^ This microRNA targets both Cnx43 and KCNQ1,^11^, ^13^, ^14^, ^40^, ^41^ and, when down‐regulated, improves KCNQ1 expression in H9c2 cells,^14^ in line with the results achieved here. Again, this is the first time that a study reports miR‐1 involvement in a telmisartan‐induced cardioprotection. One hypothesis that could be formulated for this phenomenon is that the miR‐1 down‐regulation could be linked to the blocking activity of telmisartan on the AT1 receptors, which notoriously stimulate the expression of cellular miRNAs under hypoxia conditions.^46^, ^47^ Induced by hypoxia, the AT1 receptors promote miRNA expression through mechanisms depending on Gaq/11 and Erk1/2 activation and by blocking AT1 receptors, telmisartan could impair the activation of Gaq/11 and Erk1/2 G‐protein‐dependent signalling, leading to a reduced miR‐1 expression.^46^, ^47^ On another note, telmisartan‐induced miR‐1 down‐regulation also correlated with increased viability of hypoxic/reoxygenated H9c2 cells and increased expression of the anti‐apoptotic protein Bcl‐2 into the cells.

Summarizing these points, telmisartan cardioprotected from myocardial ischaemia/reperfusion injury by modifying the cardiac levels of the three factors Cnx43, KCNQ1 and miR‐1. What still required deeper evaluation were the cell types which telmisartan‐activated and the sequence of events. It is opinion that, although non‐cardiomyocyte cells of the heart express Cnx43 gap junctions and are able to influence cardiac electrophysiology, KCNQ1 channels are a predominant feature of cardiac myocytes.^48^, ^49^, ^50^ Similarly, it is opinion that there should be a coordinated and sequential action by telmisartan on the factors monitored, probably through miR‐1 first and then on the other two. Therefore, in order to investigate a possible Cnx43/KCNQ1 modulation afforded by telmisartan through miR‐1 regulation, we translated the research on cultured hypoxic/reoxygenated embryonic rat H9c2 cardiomyocytes exposed to the hypoxia‐mimetic chemical cobalt chloride (CoCl2), and treated with telmisartan. Ventricular H9c2 cells are a well‐known relevant cell model in the mimicking of IR injury: Being the closest cells to primary cardiomyocytes concerning their energy metabolism features, such as number and arrangements of mitochondria and presence of beta‐tubulin II,^51^, ^52^, ^53^ H9c2 cells are a simple validated and useful in vitro model for exploring the mechanisms driven by hypoxia/reoxygenation.^23^, ^24^, ^25^, ^54^, ^55^ These lead to the hydroxylase enzyme inactivation, hypoxia‐inducible factor HIF‐1α stabilization, ROS generation, apoptosis and increased Bax/Bcl‐2 ratio followed by activation of caspase‐3 cleavage.^55^, ^56^

The data obtained in the present study on H9c2 cells confirmed the reduction in Cnx43, KCNQ1 and Bcl‐2 expression following exposure to the hypoxia‐mimetic agent, while treatment with telmisartan restored them. This in line with other experiences showing that angiotensin II (ATII) exerts an inhibitory effect on Cnx43, KCNQ1 and Bcl‐2 expression, and PPAR‐*γ* agonism is effective in preventing the reduction in Cnx43 induced by ATII.^57^, ^58^, ^59^ Interestingly, the telmisartan‐activated response on Cnx43, KCNQ1 and Bcl‐2 exerted through a down‐regulation of miR‐1 was confirmed by mimic transfection of miR‐1. Thus suggesting in vivo crosstalk between Cnx43, KCNQ1, Bcl‐2 and miR‐1,^3^ one would argue that the properties exerted by telmisartan in hypoxic/ischaemic cardiac damage are exerted through miR‐1 and consequent modifications of Cnx43, potassium channel KCNQ1 and Bcl‐2 expression in the cardiomyocytes.

## CONFLICT OF INTEREST

The authors confirm that there are no conflicts of interest.

## AUTHOR CONTRIBUTION

MCT performed the research, FB performed immunofluorescence, AM analysed the data, IP performed the immunohistochemical analysis, EG and GP performed the surgical procedure, and GFN and MD designed the research and wrote the study.

## Data Availability

The authors confirm that the data supporting the findings of this study are available within the article.
